# Thermoneutral environment improves mouse welfare and reduces stress in metabolic cages

**DOI:** 10.1038/s41684-025-01618-0

**Published:** 2025-10-10

**Authors:** Philipp Villiger, Charlotte Calvet, Eva Maria Pastor-Arroyo, Carsten A. Wagner, Petra Seebeck

**Affiliations:** 1 Zurich Integrative Rodent Physiology, Institute of Physiology, University of Zurich, Zurich, Switzerland; 2 Institute of Physiology, University of Zurich, Zurich, Switzerland

**Keywords:** Metabolism, Circulation, Respiration

## Abstract

Metabolic cages (MCs) are often used to collect feces and urine samples. However, the housing of mice in MCs can be stressful, potentially affecting parameters of interest. Here we compare our standard protocol for individual MC housing (4 days at 23 °C: 3 days of permanent acclimatization followed by 24 h sampling (MC23)) with a short-term intermittent acclimatization protocol (3 h of MC housing for 3 days plus 24 h sampling (accMC23)), the provision of a nest (4 days at 23 °C in MC (nest-MC23)) and MC housing at thermoneutrality (4 days at 30 °C, MC30)). C57BL6/N mice were implanted with telemetric transmitters to collect electrocardiograms, blood pressure, core body temperature and activity data. Single-housed mice in the MC at 23 °C had lower core body temperatures and higher heart and respiratory rates than mice in the MC30 group. Mice housed in MCs at 23 °C exhibited increased food consumption and weight loss, combined with significantly increased expression of messenger RNAs of key molecules in brown fat compared with mice housed in MCs at 30 °C. They also showed increased corticosterone levels. Some male mice of the MC23 and accMC23 groups exhibited episodes of reduced core body temperature and reduced blood pressure and heart rate. Our study demonstrates that housing mice in MCs at 23 °C has a substantial impact on their physiology and welfare due to a substantial cold stress. MC housing at thermoneutrality (30 °C) provides a simple solution to improve mouse welfare. Furthermore, the results showed that a single acclimatization period had the same effect as repeated exposure to the MCs and, therefore, provided no additional benefit.

## Abstract (web summary)

The study compares acclimatization protocols for metabolic cages and found that the standard housing at 23 °C impacts mice’s physiology and welfare owing to cold stress. However, thermoneutral housing provides a simple solution to improve mouse welfare.

## Main

Metabolic cages (MCs) are often used in experiments to investigate the relationship between the uptake and excretion of substances or their metabolites. A prerequisite for accurate monitoring is the reliable collection of urine and feces, while food and water intake can be monitored in parallel. To ensure continuous and individual sampling with minimal contamination, mice are housed individually on a gridded floor with no bedding, nesting or enrichment. As laboratory mice are considered to be a social species, social isolation can be assumed to act as a constant stressor^1,2^ that negatively affects physiological and metabolic parameters^1^. Single-housed mice show increased heart rate, decreased body weight^2^, decreased body temperature^3^ and more depression-like behaviors^4^. However, individual housing may be less detrimental when performed in an enriched environment such as the home cage (HC)^5–7^.

Cage enrichment has a positive effect on mouse welfare, particularly when burrowing and nesting are allowed^8,9^. Bedding and nesting materials are legally required in most European countries^10,11^ when mice are housed in their HC. Bedding, nesting and other enrichment materials are also essential for small mammals to cope with environmental challenges, as a nest allows them to regulate their thermal microenvironment according to the environmental situation, thus avoiding excessive energy loss^12–15^. Consequently, the absence of bedding and nesting materials, as well as the MC metal grid floor, prevents mice from reducing their convective heat loss^16^.

It is currently debated whether the environment temperature of laboratory mouse facilities, even in the HC environment, is too cold for mice^17^. Mice expend a substantial proportion of their energy on cold-induced thermogenesis^18^, and it has been suggested that cold stress may be one of the reasons why mouse physiology and metabolism differ from human conditions^15,19,20^. Cold stress stimulates thermogenesis by activating brown adipose tissue (BAT) and increases blood pressure, heart rate and respiratory rate.

Only a few studies have investigated MC housing in mice. These studies showed that mice housed in MCs had an elevated blood pressure for several hours and prolonged tachycardia lasting 2 days^21^ and suggested that MC housing accelerated metabolism^16,22–24^ and increased oxidative stress^16^. While some studies showed that mice had stable urination rates in the MC after 3–4 days^25^, others found that mice did not acclimatize^21,22,26^, even after 3 weeks^16^. Acclimatization was associated with an increase in food/water intake and urine output^25,27^. Increased food intake may indicate increased thermogenesis as evidenced by upregulation of *Ucp1* expression in BAT^22^ and subsequent loss of fat^22–24^ or body mass^16,22–27^. Another study showed increased urinary concentrations of norepinephrine, vanillylmandelic acid and corticosterone^26^ and an increase in anxiety-like behavior when mice were housed alone in MC^26^.

Commonly used protocols include several days of continuous or intermittent acclimatization before sampling. MCs are maintained under standard housing room temperature (ambient temperature of 20–24 °C). However, the contribution of ambient temperature as a major stressor remains largely unclear^16,21–27^, whereas a recent study demonstrated a beneficial effect of thermoneutral temperature on metabolic parameters^24^. To our knowledge, only one study has measured body temperature in mice during MC housing, showing an average body surface temperature of approximately 30 °C at an ambient cage temperature of 22.5 °C (ref. ^22^). A recent study showed increased corticosterone levels and energy expenditure in MCs at standard temperature compared with thermoneutral conditions^24^. In addition, the effect of stress has been related to temperature-dependent changes in intestinal motility^28^, suggesting that too low temperatures can cause stress leading to measurable physiological changes.

Hypothesizing that mice in MC experience cold stress at standard temperature, we aimed to determine the stress level of a standard MC housing regime and compare it with three different potential enhancements. We modified either the acclimatization regime, the environment temperature or provided a nest with nesting material. All groups contained male and female mice and were compared with the single HC housing situation.

We used longitudinal synchronized telemetry and video recordings to comprehensively assess and evaluate physiological and behavioral data. Mice were implanted with telemetry transmitters to continuously measure core body temperature, mean arterial blood pressure, heart rate, respiratory rate and activity. Telemetry activity data were analyzed for total activity and video data for the time spent in specific cage areas. We also quantified food and water intake, feces and urine output, urinary stress hormones, electrolytes and proteins. To assess the difference in thermoregulatory stress between MC housing at 23 °C and 30 °C, we analyzed changes in body composition, ketone body levels and gene expression in the BAT of two groups without transmitter implantation.

## Results

The animals were divided into four different groups:

1. Standard MC housing at 23 °C for 4 days (MC23)
2. Three days of short-term (3 h) intermittent acclimatization, followed by 24 h of permanent MC housing (accMC23)
3. MC housing at 23 °C for 4 days with nest and nesting material (nest-MC23)
4. MC housing at 30 °C for 4 days (MC30)

### Highest body weight loss with the standard MC housing conditions

**Fig. 1:**
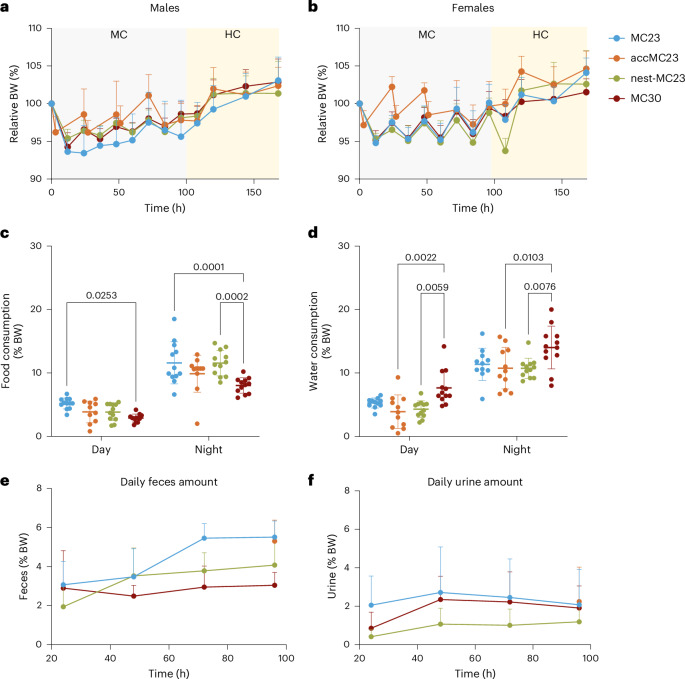
Changes in body weight of mice housed in MCs under different conditions. **a**,**b**, Relative body weight (BW) of males (**a**) and females (**b**) normalized to initial body weight at the start of MC housing (mean + s.d.). **c**,**d**, Relative food (**c**) and water intake (**d**), respectively, normalized to body weight during the sampling day of the MC housing period (day 4) (mean ±  s.d.). Statistics were performed with two-way ANOVA followed by Šídák’s multiple comparison test (the *P* values are indicated in the graphs). **e**,**f**, Total daily collected feces (**e**) and urine (**f**) normalized to body weight (mean + s.d.) For the accMC23 group, only the sampling day (day 4) of MC housing was considered. Single-housed mice were either continuously kept in MCs at 23 °C for 4 days (MC23, blue, *n* = 6 female (♀) + 6 male (♂)), acclimatized for 3 h per day until day 4 (accMC23, orange, *n* = 5♀ + 6♂) or provided with a nest (nest-MC23, green, *n* = 6♀ + 6♂). Another group was housed in a thermoneutral environment in the MC at 30 °C (MC30, red, *n* = 6♀ + 6♂). The experiment was conducted in ten batches depending on animal availability and groups.

During the 4 days in the MC, all male mice of all groups lost body weight. Even the accMC23 group male mice lost up to 5% body weight daily when they were placed in the MC for 3 h (Fig. 1a,b). Not all mice of both sexes of the MC23, nest-MC23 and MC30 groups recovered their initial body weight while housed in the MC. However, the mice recovered body weight when they were returned to their HC (Fig. 1a,b)—a pattern already seen in the accMC23 group during the acclimatization. Males lost more and recovered less of their initial body weight during MC housing compared to females (Fig. 1a,b). Daily food intake increased over time in all groups (Supplementary Fig. 1a), except the MC30 group, eating significantly less than the MC23 group (*P* = 0.0253 daytime and *P* = 0.0001 during night, two-way analysis of variance (ANOVA) with Šídák’s multiple comparison test) (Fig. 1c). By contrast, water intake was increased only under thermoneutral conditions (*P* = 0.022 during day and *P* = 0.0103 during night) (Fig. 1d and Supplementary Fig. 1b). Feces excretion tended to increase from day 1–4 during MC housing in the MC23 and the nest-MC23 groups but remained constant under thermoneutral conditions (Fig. 1e). On day 4 in MC, animals in the MC30 group excreted significantly less feces than the other groups (*P* = 0.0037) (Supplementary Fig. 1c). For mice in the nest-MC23 group significantly less feces was collected during the day (*P* = 0.0063) compared with MC23 (Supplementary Fig. 1c). The amount of collected urine varied over the course of the experiment in all groups. In the nest-MC23 group, significantly less urine was collected in at day 5 morning compared with the other groups (*P* = 0.0404) (Fig. 1f and Supplementary Fig. 1d), which was mainly due to male mice urinating inside their nest.

### Increased electrolyte excretion in the MC23 and accMC23 groups

**Fig. 2:**
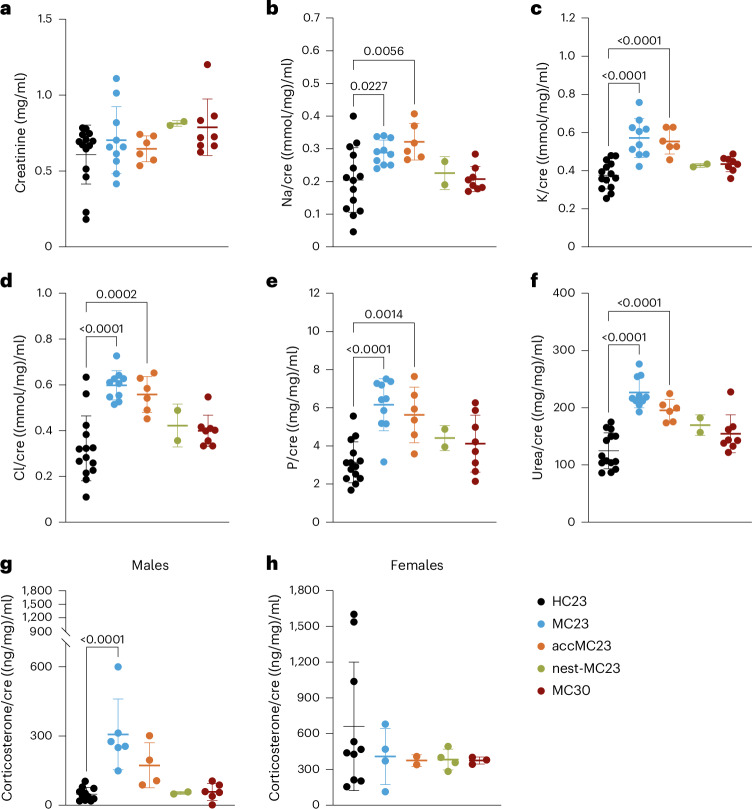
Changes in urinary electrolyte and corticosterone concentrations. **a**–**f**, Creatinine (**a**), sodium (**b**), potassium (**c**), chloride (**d**), phosphorus (**e**) and urea (**f**) concentrations normalized to creatinine night urine samples. **g**,**h**, Urinary corticosterone concentration normalized to creatine excretion in males (**g**) and females (**h**). A one-way ANOVA with Dunnett’s multiple comparison test was used for all graphs (the *P* values are indicated in the graphs) (*n* = 2–11). In **a**–**h**, the data are shown as ±s.d. Single-housed mice were either continuously kept in MCs at 23 °C for 4 days (MC23, blue, *n* = 6♀ + 6♂), acclimatized for 3 h per day until day 4 (accMC23, orange, *n* = 5♀ + 6♂) or provided with a nest (nest-MC23, green, *n* = 6♀ + 6♂). Another group was housed in a thermoneutral environment in the MC at 30 °C (MC30, red, *n* = 6♀ + 6♂). The experiment was performed in ten batches, depending on the availability of animals and groups. Morning urine (19:00–7:00) of the sampling day (day 4) was used for measurements. The baseline spot urine samples were collected from all groups in the morning before the start of baseline (HC23) measurements.

For all groups MC urinary creatinine levels were not different from baseline (HC23) samples (Fig. 2a). Overnight urinary electrolyte excretion normalized to creatinine was significantly increased in the MC23 and accMC23 groups compared with HC23 (Fig. 2b–f). Differences (all analyzed with a one-way ANOVA with Dunnett’s multiple comparison test) compared with HC23 were observed for sodium (*P* = 0.0227 for MC23 versus baseline and *P* = 0.0056 for accMC23 versus baseline, respectively), potassium (*P* < 0.0001 for MC23 versus baseline and *P* < 0.0001 for accMC23 versus baseline, respectively), chloride (*P* < 0.0001 for MC23 versus baseline and *P* = 0.0002 for accMC23 versus baseline, respectively), phosphorus (*P* < 0.0001 for MC23 versus baseline and *P* = 0.0014 for accMC23 versus baseline, respectively) and urea (*P* < 0.0001 for MC23 versus baseline and *P* < 0.0001 for accMC23 versus baseline, respectively) (Supplementary Fig. 2a). Urinary magnesium was increased in the MC23 (*P* = 0.0183) and accMC23 (*P* = 0.0119) groups compared with baseline, whereas calcium and albumin were increased only in the accMC23 group (*P* = 0.0296 and *P* = 0.0114, respectively) (Supplementary Fig. 2b–d). In addition, corticosterone levels were 6.4-fold and 3.6-fold higher in male MC23 compared with male HC23 (*P* < 0.0001) and male accMC23 (*P* > 0.05) mice, respectively (Fig. 2g). HC23 corticosterone levels were higher and more variable in females than in males. However, corticosterone levels showed no statistical significance between all the groups and HC23 (Fig. 2h). In conclusion, the MC23 and accMC23 groups showed altered urinary electrolyte excretion compared with HC23 in both sexes, but corticosterone levels were only increased in MC23 males. The MC30 and nest-MC23 groups showed no differences in electrolyte and corticosterone levels compared with HC23.

### Reduced core body temperature in the MC23 group

**Fig. 3:**
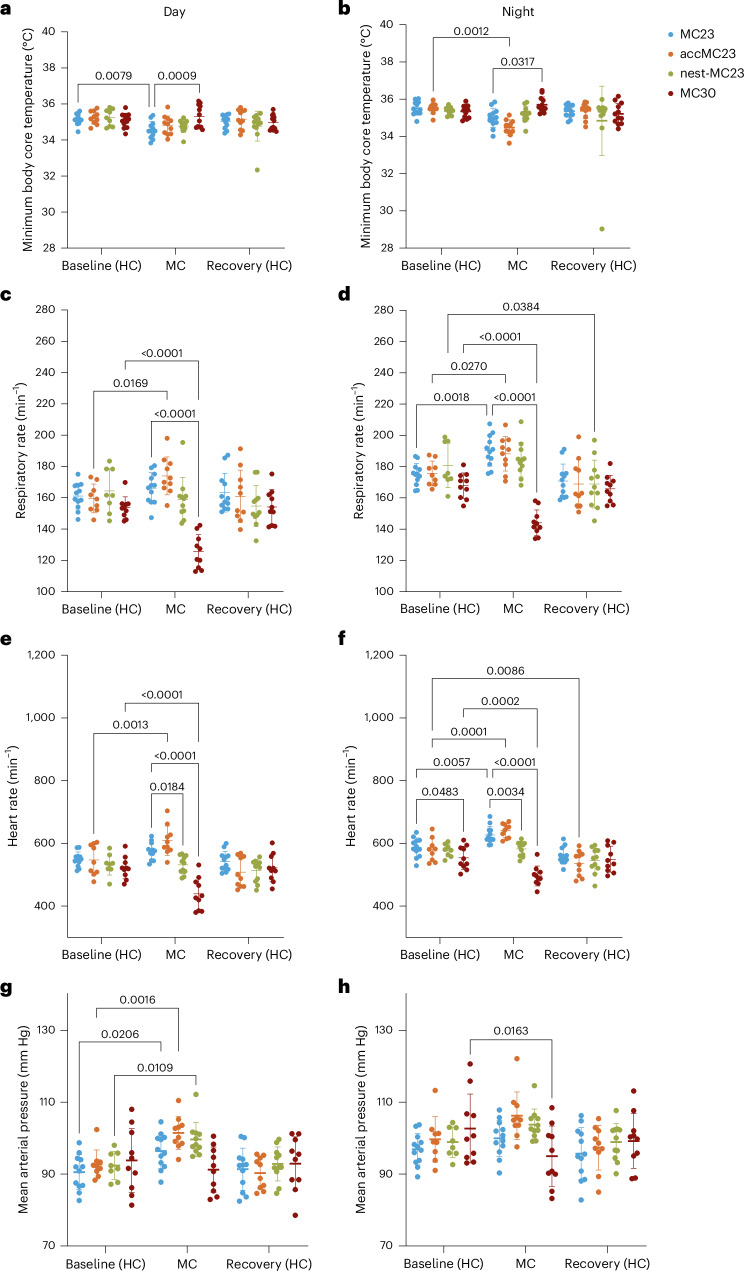
Changes in physiological parameters. **a**–**h**, Physiological parameters measured during the day (**a**, **c**, **e** and **g**) and during the night (**b**, **d**, **f** and **h**) using an abdominal implanted HD-S11 transmitter. For the baseline and recovery periods, the entire 48 h and 72 h, respectively, were considered; for the MC, only data from the sampling day (24 h/day 4) are shown. The parameters include lowest core body temperature (**a** and **b**), mean respiratory rate (**c** and **d**), mean heart rate (**e** and **f**) and mean arterial blood pressure (**g** and **h**). Two-way ANOVA followed by Dunnett’s multiple comparison test was performed for all groups (the *P* values are indicated in the graph) (*n* = 9–12). In **a**–**h**, the data are shown as ±s.d. Single-housed mice were either continuously kept in MCs at 23 °C for 4 days (MC23, blue, *n* = 6♀ + 6♂), acclimatized for 3 h per day until day 4 (accMC23, orange, *n* = 5♀ + 6♂) or provided with a nest (nest-MC23, green, *n* = 6♀ + 6♂). Another group was housed in a thermoneutral environment in the MC at 30 °C (MC30, red, *n* = 6♀ + 6♂).

At baseline and during recovery, the minimum core body temperature, respiratory rate, heart rate and mean arterial blood pressure did not differ between groups (Fig. 3 and see Supplementary Fig. 3 for details).

During MC housing, the minimal core body temperature was significantly reduced in the MC23 group compared with baseline conditions (*P* = 0.0079; two-way ANOVA followed by Dunnett’s multiple comparison test) (Fig. 3a). In addition, the MC23 group had a significantly lower mean core body temperature than the MC30 group (*P* = 0.0009 (day) and *P* = 0.0317 (night), respectively) (Fig. 3). One single animal in the nest-MC23 group experienced a fall in core body temperature below 32 °C during the day and night periods of the recovery phase for unknown reasons (Fig. 3a,b). The respiratory rate was reduced during the day and night in the MC30 group (*P* < 0.0001), compared with baseline conditions and compared with MC23. By contrast, the respiratory rate was significantly increased in the MC23 (night) and accMC23 (day and night) groups, compared with baseline conditions (see Fig. 3c,d for significances). Similarly, the heart rate was significantly increased in the accMC23 group (*P* = 0.0013) and decreased in the MC30 group (*P* < 0.0001) during the day compared with baseline conditions (Fig. 3e). During the night, the heart rate increased in the MC23 (*P* = 0.0057) and accMC23 (*P* = 0.0001) groups compared with baseline conditions, whereas it decreased in the MC30 group (*P* = 0.0002) (Fig. 3f). The heart rate of the nest-MC23 group remained unchanged during MC housing compared with baseline conditions (Fig. 3e,f). Mean arterial blood pressure was elevated in animals in the MC23 (*P* = 0.0206), accMC23 (*P* = 0.0016) and nest-MC23 (*P* = 0.0109) groups during the inactive phase compared with baseline (Fig. 3g). In the MC30 group, blood pressure was decreased during the active phase compared to baseline (*P* = 0.0163) (Fig. 3h).

In summary, the MC30 group was the only group that did not show an increase in physiological parameters compared with their baseline (HC23 values), in contrast to the other groups.

### No habituation to MCs during short-term intermittent acclimatization

We used a short-term intermittent acclimatization schedule in which the mice were placed in the MC for 3 h on three consecutive days and then returned to their HC for the following 21 h. However, despite repeated placement in the MC, the core body temperature, heart rate and mean arterial blood pressure increased in the accMC23 group each time they were placed in the MC from their HC (Supplementary Fig. 4). Mean arterial blood pressure and the heart rate always returned to the same level again when placed back into their HC (pressure: ~120 mmHg, heart rate: up to ~800 bpm) (Supplementary Fig. 4). This increase in vital parameters was greater when placed in the MC than compared with being place in the HC (Supplementary Fig. 5). In addition, all physiological parameters were higher in the accMC23 group compared with the MC23 group elevated during MC housing on the day of sampling (Supplementary Fig. 6).

### No increased activity in the MC when mice had a nest

**Fig. 4:**
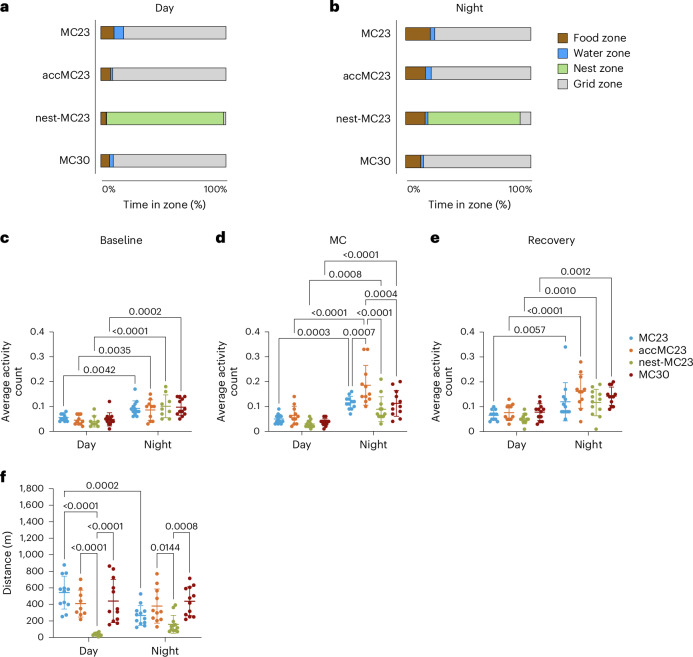
Video-tracking analysis of mice in MCs under different conditions. **a**,**b**, A percentage of time spent in each zone (food zone, water zone, nest zone, on the grid) of the MC during the day (**a**) and night (**b**). **c**–**e**, The average number of activity counts (arbitrary unit) measured by the implanted HD-S11 transmitter during baseline (**c**), MC housing (**d**) and recovery (**e**). **f**, The total distance traveled during 12 h of MC housing (day: 7:00–19:00, night: 19:00–7:00). A two-way ANOVA with Šídák’s multiple comparison test was used to compare groups in **c**–**f** (the *P* values are indicated in the graph, *n* = 9–12). The videos were recorded and analyzed with Ethovision XT in **c**–**f**. For video analysis, only videos from the sampling day (day 4) were used. In **c**–**f**, the data are shown as ±s.d. For **a** and **b**, only the mean is shown. Single-housed mice were either continuously kept in MCs at 23 °C for 4 days (MC23, blue, *n* = 6♀ + 6♂), acclimatized for 3 h per day until day 4 (accMC23, orange, *n* = 5♀ + 6♂) or provided with a nest (nest-MC23, green, *n* = 6♀ + 6♂). Another group was housed in a thermoneutral environment in the MC at 30 °C (MC30, red, *n* = 6♀ + 6♂). The experiment was performed in ten batches according to the availability of animals and groups.

Mice from the MC23, accMC23 and MC30 groups preferred to stay close to the food hopper (Supplementary Fig. 7). All mice also spent more time in the food and water zones during the night than during the day. Mice provided with a nest in the MC spent most of their time in the nest (day: 89.7%, night: 67.5%) (Fig. 4a,b). During MC housing, we observed an increased activity in the accMC23 group compared with the nest-MC23 (*P* ≤ 0.0001, Two-way ANOVA with Šídák’s multiple comparison test) and MC30 (*P* = 0.0004) groups during the night (Fig. 4d). This increased activity disappeared during the recovery period (Fig. 4e). Nevertheless, all mice maintained their original day/night rhythm, according to their activity and food/water intake patterns throughout the experiment (Figs. 1a–d and 4c–e). However, the different groups showed large differences in the distances traveled (Fig. 4f). During the day, the nest-MC23 mice did not move much (39 ± 17 m), whereas mice from the MC23, accMC23 (*P* < 0.0001) and MC30 (*P* < 0.0001) groups moved more than a tenfold greater distances: 543 ± 199 m, 405 ± 154 m and 441 ± 261 m, respectively. During the night, mice from the nest-MC23 group also traveled significantly less (159 ± 109 m) than the accMC23 and MC30 groups: the accMC23 group traveled 374 ± 198 m (*P* = 0.0144 versus nest-MC23) and the MC30 group 438 ± 174 m (*P* = 0.008).

### Ketone body concentration and genes regulating BAT increased at 23 °C

**Fig. 5:**
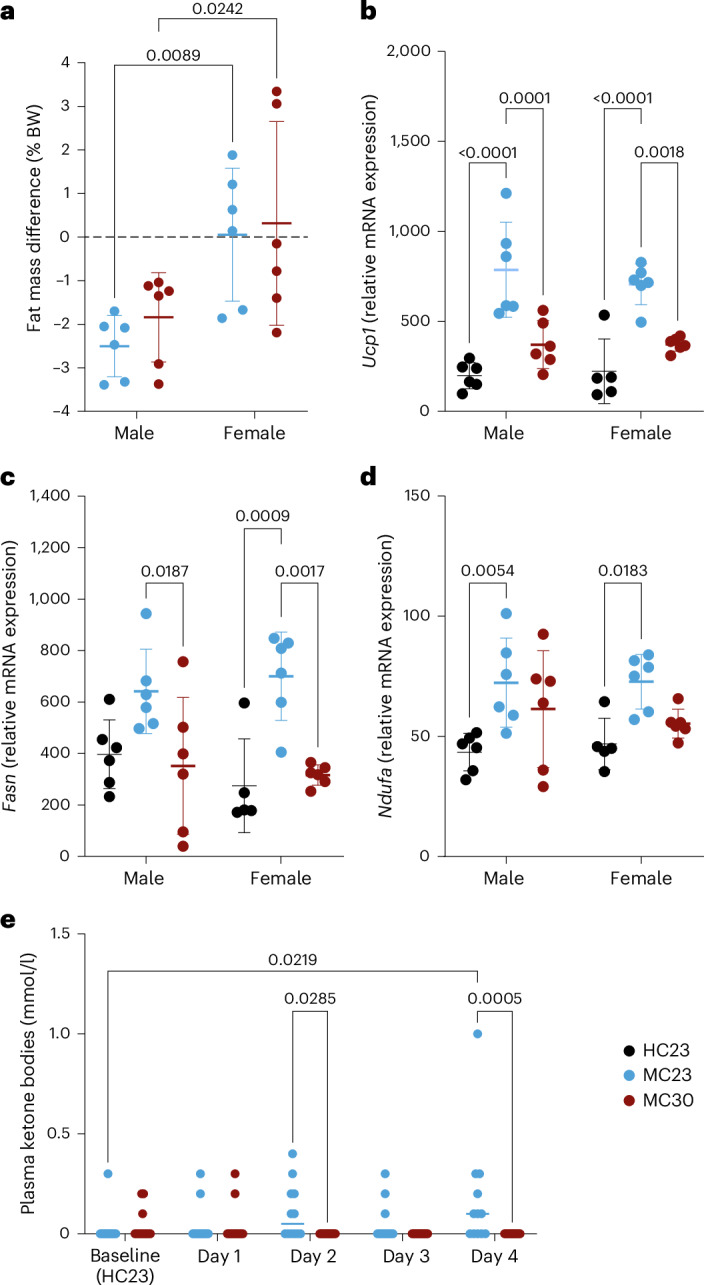
Changes in body composition and ketone body concentration. **a**, Body composition was measured in nonimplanted mice before MC housing (HC23) and at the end of MC housing. **b**–**d**, Relative gene expression of *Ucp1* (**b**), *Fasn* (**c**) and *Ndufa* (**d**) in BAT. BAT was collected immediately after echo MRI measurements. **e**, Ketone body concentration measured in a blood sample obtained by tail-vein puncture. The concentration was measured daily at 16:00 h while the mice were kept in a handling tube. A one-way ANOVA with uncorrected Fisher’s least significant difference test in **a** and two-way ANOVA with Tukey’s multiple comparisons in **b**–**e** statistical tests were performed (the *P* values are indicated in the graph) (*n* = 5–6). In **a**–**e**, the data are shown as ±s.d. Single-housed mice were either kept in MC at 23 °C for 4 days continuously (MC23, blue, *n* = 6♀ + 6♂) or in a thermoneutral environment in the MC at 30 °C (MC30, red, *n* = 6♀ + 6♂). Average of all differences in fat mass was calculated from the measured fat ratios before and after MC housing.

At both temperatures (23 °C and 30 °C), only male mice lost fat (−1.8 to −2.5% body weight), whereas the females maintained their fat content constant (+0.06 to +0.31% body weight) (Fig. 5a). By contrast, gene expression analysis in the BAT revealed no sex differences in the mRNA expression of *Ucp1*, *Fasn* and *Ndufa*, normalized to the housekeeping gene *HPRT* (Fig. 5b–d). *Ucp1* was elevated in the MC23 group (males by 3.96-fold, *P* < 0.0001, females 3.17-fold, *P* < 0.0001; two-way ANOVA with Tukey’s multiple comparisons test) compared with the baseline HC23 conditions. The MC30 group had significantly lower levels of *Ucp1* than the MC23 group (males: 2.12-fold increase, *P* < 0.0001; females: 1.89-fold increase, *P* = 0.0018). We observed increased expression of *Fasn* and *Ndufa* in the MC23 group compared with MC30 in both males (*P* = 0.0187) and females (*P* = 0.0017) but no difference between the MC30 group and HC23 conditions. In addition, we observed that the blood ketone body concentration increased in some mice up to 1 mmol/l in both sexes over the 4 days in the MC23 group, whereas it remained below the detection limit of 0.1 mmol/l in all mice of the MC30 group from day 2 onwards (Fig. 5e).

### Male mice housed at standard temperature showed episodes of reduced cardio-metabolic activity

**Fig. 6:**
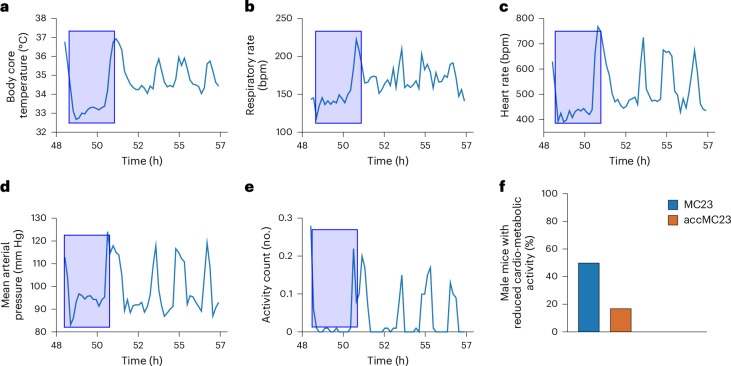
Episodes of reduced cardio-metabolic activity in mice housed in MCs at 23 °C. **a**–**e**, Time-locked telemetry data (hours 48–57 of the experiment) from a male mouse of the MC23 group while housed in the MC, which include core body temperature (**a**), respiratory rate (**b**), heart rate (**c**), mean arterial blood pressure (**d**) and activity (**e**) measured with an abdominally implanted HD-S11 transmitter over the course of 8 h. The blue rectangles indicate a 2-h episode. **f**, The percentage of male mice in each group showing at least one episode of reduced cardio-metabolic activity during MC housing. Single-housed mice were either continuously kept in MCs at 23 °C for 4 days (MC23, blue, *n* = 6♀ + 6♂) or acclimatized for 3 h per day until day 4 (accMC23, orange, *n* = 5♀ + 6♂).

We observed episodes of apparent reduced cardio-metabolic activity in male mice of the MC23 and the accMC23 groups but not in any female mouse. These episodes were characterized by the concomitant decrease in core body temperature, respiratory/heart rate and mean arterial blood pressure for at least 120 min compared with the previous individual data. In addition, the mice had to be inactive. Figure 6a–e shows an example for an individual mouse with core body temperature: −8%, heart rate: −21%, respiratory rate: −11%, mean arterial blood pressure: −10%. In the MC23 group, 50% of the male mice showed at least one such episode and in the accMC23 group, one in six male mice (Fig. 6f). One male MC23 mouse even had three episodes during the experiment. By contrast, no such events were observed in the nest-MC23 and the MC30 groups.

## Discussion

We aimed at providing a comprehensive and multifaceted view of the effects of MC housing on mouse welfare and therefore used a combination of different techniques. We hypothesized that mice housed in MCs at standard housing temperature would experience substantial cold stress as suggested by others^23,24^. Heart rate and blood pressure are known to increase during stressful situations such as restraint^29^. Food intake, body composition, core body temperature, BAT markers, ketone bodies and activity patterns can be used to describe the acclimatization to different environmental conditions or the metabolic state of the animal. Stress hormone analyses are commonly used to detect stress in rodents^2,5,16,22,24,26^; however, they cannot provide the same temporal resolution as continuous undisturbed monitoring of physiological parameters. Corticosterone is a slow acting parameter that allows the detection of chronic stress^16^.

### Mice housed in MC at standard temperature (23 °C) experience cold stress

A recent study showed that mice housed in MC at 23 °C without a nest experienced substantial cold stress, as evidenced by an upregulation of their energy expenditure^24^. Small mammals exposed to cold increase their heat production in combination with a reduction in energy loss (for example, by insulating themselves with nest material). Heat production requires energy, and mice kept in colder environments eat more than those kept in warmer environments^14^. This may explain the increased food intake over time observed in the MC23 group but not in the MC30 group. In line with recent research^30^, we showed that exposure of MC23 mice to below thermoneutral temperatures resulted in increased blood pressure, heart and respiratory rate, whereas the MC30 group did not show this increase.

Despite increased food intake, laboratory mice lose substantial body weight when transferred to colder environments^14^, presumably due to fat loss. As described above, we observed a body weight loss in all groups during MC housing, although the weight loss was much lower than that reported in other studies^22–27^ (a weight loss of up to 27% in male and 21% in female mice^27^). This difference might be explained by the fact that we used younger mice with ad libitum access to food and/or gentle (tube) handling. The mice housed in MC at 23 °C ate more but still lost more body weight than those housed at 30 °C. As reported previously, we observed a loss of fat mass after 4 days of MC housing under both standard (23 °C)^22–24^ and thermoneutral (30 °C)^24^ conditions in male but not in female mice. We also observed an increase/stabilization of feces and urine excretion over the 4 days of MC housing at 23 °C^16,23,25,27^. By contrast, food consumption and feces excretion remained constant over the 4 days in the MC30 group, indicating a stable caloric requirement comparable with the HC environment.

The mice housed in MC spend most of their time in the area of the food hopper^23^. Our study confirmed this finding, since in all groups (except the nest-MC23) the mice spent most of their time near to the food hopper. We therefore suggest that this finding is not related to the food in the hopper itself but rather indicates that the hopper is the most ‘protective’ refuge, except if a nest is available.

Small mammals increase their thermogenesis mainly by activating the BAT. *Ucp1* is the major protein that mediates thermogenesis by uncoupling the ATP production from the oxidative phosphorylation in the mitochondria of BAT adipocytes^31^. Consistently, a strong upregulation of *Ucp1* expression in mice housed in MCs at 23 °C has been reported by others^22^. We confirmed that nonshivering thermogenesis in the BAT may be upregulated in MC23 mice compared with the HC23 housing but not in MC30 mice. In addition to *Ucp1*, *Fasn* and *Ndufa*, two genes essential for maintaining the oxidative respiration in the mitochondria, were also upregulated in the MC23 group compared with HC23.

In our study, mice housed in MC at 23 °C had a significantly lower core body temperature compared with baseline conditions, whereas mice housed in MC at 30 °C had a higher core body temperature than MC23 mice. This is a common mechanism by which mice lower their skin temperature to reduce radiative heat loss^14^. In addition, the body switches to ketogenesis when energy is scarce. Normally, ketones are only produced during aperiods of food restriction or starvation, mainly by fatty acid oxidation in the liver^32^. We observed significantly elevated ketone blood levels in the MC23 group compared with the MC30 group. These elevated ketone levels suggest that MC23 mice, even when given ad libitum access to food, switched metabolically to increased fat oxidation. Moreover, some mice in the MC23 and accMC23 groups showed episodes of reduced cardio-metabolic activity characterized by a fall in core body temperature together with a reduction in heart rate, blood pressure and respiratory rates, suggesting that these mice experienced heightened stress.

Studies evaluating the stress levels in mice housed in MC have reported elevated corticosterone concentrations^16,22,24,29^. In our study, the corticosterone levels were elevated in MC23 mice but not in the MC30 mice compared with levels in the HC23 conditions. However, in contrast to others^24^, we observed increased corticosterone levels only in male mice. Female mice showed a high variability of corticosterone levels before and during MC housing, which does not allow to reach a clear conclusion. Studies analyzing the stress response in rodents via the hypothalamic–pituitary–adrenal axis have shown that there may be sex differences^33^. A possible explanation for the higher variability in the females could be differences in their individual estrous cycle state, which we did not test for^34^.

Finally, urine concentration did not differ between the groups, but the increased food intake resulted in increased excretion of electrolytes, minerals and proteins in the MC23 and accMC23 groups compared with baseline HC23. These changes in urinary biochemistry need to be taken into account when comparing MC data with the standard HC situation and when describing genetically modified/aging animals or progressive disease models.

### Short-term intermittent adaptation did not reduce stress

Although gentle (tube) handling was used, we observed a short-term transient increase in blood pressure and heart rate during the health checks, most likely related to handling stress^29^. However, in the accMC23 group, we observed a higher repetitive increase in both parameters after each placement in the MC, suggesting that these mice were equally stressed by the acclimation procedure on all four consecutive days and did not show signs of acclimation to the MCs. We were also able to show that on day 4 all stress-related physiological parameters (such as the respiratory rate, blood pressure and the heart rate) were higher or equally high as the initial values measured in mice from the MC23 group on their first day of MC housing. In addition, we measured a 3.6-fold increase in urinary corticosterone excretion in the males of the accMC23 group compared with HC23.

In conclusion, intermittent short-term acclimation did not result in a measurable stress-reducing effect. As the response was less intense when the mice were transferred to their HC, we suggest that the stress was not exclusively related to the handling and environmental change per se but specifically related to the MC environment. The observed increase is consistent with a study reporting a transient increase in mean arterial blood pressure and heart rate after placing mice in MCs^21^.

### A nest interfered with sample collection

Nest-MC23 mice used their nest and spent up to 89% of their time in it. Compared with the MC23 group, these mice did not have an elevated blood pressure, heart rate or respiratory rate, despite being housed at the same ambient temperature of 23 °C. Moreover, the mean and maximum core body temperatures were comparable with the physiological ranges at baseline (HC23 values). Corticosterone levels were also not elevated compared with HC23. Taken together, these mice benefitted from the nest. However, significantly less urine was collected from this group. Male mice, in particular, tended to urinate in the nest and therefore, the amount of urine collected was insufficient for all analyses. This renders the nests unsuitable for MC experiments and perfectly illustrates the conflict between scientific and welfare needs: the best refinement for the mice does not always give the best results for the scientific experiments.

Researchers use rodents as models of human disease to mimic the human organism. However, even in the HC environment, the ambient housing temperature of laboratory mouse facilities can be too cold for mice^17^. It has been suggested that this may be one of the factors contributing to the inadequacy of mouse physiology and metabolism in mimicking the human condition^15,19,20^. We confirm that housing mice in MC at 23 °C adds an (unwanted) cold stress to the experiment, which could be even more detrimental for fragile animals such as confined, genetically modified, aging animals or models of progressive diseases. This is especially true when the data obtained are compared with other cohorts in other housing environments. We have also shown that raising the ambient temperature to thermoneutrality (30 °C) is a simple solution to improve both the welfare of the mice and the quality of the data. Housing mice in MC at 30 °C substantially improved the situation, avoided energy starvation episodes and substantially increased core body temperature of the mice without presumably increasing metabolic rate. This is consistent with previous recommendations for other experiments to house mice at thermoneutrality to improve welfare and translatability to humans^15,18,19,35,36^.

We demonstrate that intermittent short-term acclimatization provides no additional benefit. Rather than using (permanent or intermittent) acclimatization protocols of several days’ duration, we suggest that mice should be placed in MC for (half) a day before a 24-h sampling period to permits sufficient acclimatization to the novel environment, ideally at 30 °C.

## Conclusion

Our study shows that housing mice in MCs at standard housing temperature does not reproduce the physiology of mice in standard HC housing and that mice housed in MCs at 23 °C are subjected to a substantial cold stress that accelerates and alters their metabolism. In conclusion, mice housed in MCs at 23 °C lost more body weight despite eating more, showed higher heart and respiratory rates in combination with a lower body core temperature, a strongly activated thermogenesis and episodes of reduced cardio-metabolic activity compared with mice housed in MCs at 30 °C. Furthermore, our results support the notion that female mice may have a greater capacity for adaptive responses to change and may be better able to cope with challenging environments, highlighting the importance of using both sexes in experiments.

## Methods

### Ethical statement

All animal experiments were designed according to the PREPARE/ARRIVE guidelines and approved by the Swiss cantonal veterinary authorities (licence numbers ZH212/2021 and ZH129/2021).

### Animals

A total of 72 C57BL6/N mice (36 male and 36 female) (Charles River, Germany), aged 14–20 weeks were used. The mice were habituated to the facility environment for at least 10 days before the experiment and were handled with tunnels.

### Housing

Before surgery, animals were housed in groups of two to four in standard IVC cages (NexGen Mouse500) with wood chips, shredded paper, paper tissues, a red translucent plastic house and a transparent plastic tunnel. The mice had ad libitum access to food (Kliba-Nafag product no. 3436, GRANOVIT AG) and water. Room temperature was maintained at 22–24 °C and the relative humidity at approximately 50% during a 12–12 h light–dark cycle with lights on at 7:00 h. After surgery, the mice were housed at least in pairs in a climate chamber (UniProtect Luftstromschrank, Zoonlab GmbH), which was warmed to 30 °C for 4 days after surgery and then reduced to 23 °C thereafter. The animals were transferred to customized cages (art. no. 100188, Verutech AG) at least 1 day before the baseline period. At this time, the mice were housed individually. For the recovery period after MC housing mice were again housed in the same cages. During the MC housing period, the mice were housed in a mouse MC (art. no. 3600M021, Tecniplast), equipped with a mouse arch (Plexx). All cages were placed in the climate chamber to maintain a constant ambient temperature. In the MC, the mice had ad libitum access to their standard powdered chow and water.

### Surgery/recovery

A total of 24 male and 24 female C57BL6/N mice were implanted with HD-X11 transmitters (Data Science International) under isoflurane anasthesia and appropriate analgesia (5 mg/kg carprofen, preoperatively and twice daily for 3 days postoperatively, while maintained on fluids (1 ml subcutaneous warmed ringer’s lactate), intensive care nutrition (glucose soaked standard chow pellets)^37,38^. Surgery was performed as previously described by others^39,40^.

### Study design

All implanted mice were individually housed in their HC for 48 h to collect baseline data, followed by 96 h in MCs according to their study group schedule. For the accMC23 group, the first 3 days were considered as an acclimatization period (days 1–3) and the fourth day as the sampling day (day 4). For all groups, the analysis was performed on the data of the sampling day, which was identical for all groups. On the fifth morning of the study, all implanted mice were returned to HCs for a 72 h recovery period.

### Study groups

The implanted mice were divided into four different groups: MC23, accMC23, nest-MC23 and MC30 (Supplementary Fig 8). The standard MC23 group was kept in the MC at 23 °C for 4 days without interruption (except for daily checks). The accMC23 group was housed in the MCs at 23 °C but with a 3 h acclimatization schedule for 3 days (3 h MC, 21 h HC per day), followed by 24 h of continuous MC housing for sampling. For the nest-MC23 group, a three-dimensional-printed nest—initially filled with 5 g of sizzling paper—was attached to the back of a MC. As with the MC23 group, these mice were kept at 23 °C throughout 4 days. The MC30 mice were continuously housed for 4 days in the MC at 30 °C. In addition, 24 nonimplanted mice, which underwent body composition measurements before and after MC housing, were placed in the MC for 4 days at either 23 °C or at 30 °C. Throughout the study, male and female mice were housed separately but had visual, olfactory and auditory contact.

### Telemetry measurements and analysis

The mice implanted with HD-X11 transmitters were recorded throughout the experiment using Ponemah 6.51 (Data Science International). Telemetry data were reviewed using Ponemah and extracted at 10-min intervals.

### Video recording and analysis

Each cage was individually recorded using Logitech Brio 4K webcams (Logitech) running on media recorder 6.2 (Noldus). The cameras were placed on top of the HC or the MC. White light was used during the day phase and red light during the night phase. The recorded videos were cut into half-day videos (approximately 11–12 h in length) and adjusted to obtain a high-contrast greyscale video (CapCut version 2.7.0, ByteDance). Behavioral and mobility data were extracted using EthoVision XT 17.5 (Noldus).

### Urine biochemistry

For baseline (HC23) samples, spot urine was collected in the early morning and evening. MC urine samples were collected either at 7:00 f (12 h overnight urine) or at 19:00 h (12 h daytime urine) under mineral oil. Urine samples were stored at −80 °C until used for the measurements. Chloride, potassium, sodium, calcium, microalbumin, magnesium, phosphorus, urea and creatinine were measured in the overnight urine using a UniCel DxC 800 Synchron Clinical System (Beckman Coulter).

### Corticosterone measurement

A corticosterone ELISA kit (ADI-900-097, LOT 43GE160, Enzo Lifesciences) was used for quantification in urine samples (dilution 1:40) according to the manufacturer’s specifications. Absorbance values were determined using a spectral photometer (µQuant, BioTek). Extrapolation of the standard curve was performed using GraphPad Prism 10.0.3 (GraphPad Software).

### Measurement of body composition

Three consecutive measurements were taken using an EchoMRI500 whole body composition analyzer (Echo Medical Systems) and averaged per animal and per time point. Body composition was measured just before placement in the MC and just before euthanasia, immediately after the MC housing.

### Measurement of ketone bodies

The mice were held in handling tubes and punctured with a 24G needle (Terumo) in the lateral tail vein. The blood drop was collected directly onto a ketone body strip device (StatStrip Xpress2, Nova Biomedical) for measurement of ketone body concentration.

### Sacrifice

The mice were killed after measuring body composition using an automated CO_2_ station (GasDocUnit, Medres Medical Research). After cessation of respiration, blood was collected by cardiac puncture using a 24G needle (Terumo) and a 3 ml syringe (Omnifix, B. Braun SE). BAT was collected from the cervical region between the shoulder blades. The collected tissue samples were snap frozen in liquid nitrogen and stored at −80 °C until analysis.

### Gene expression analysis

The primers were designed by using the primer3 program (version 4.1.0)^41–43^ for three genes (*Fasn*, *Ndufa9* and *Ucp1*), normalized to *HPRT*. Special care was taken to select primers that overlapped an exon/exon junction, gave a product size between 80 and 150 bp, had a primer size of 18–27 bp and a *T*_m_ (melting temperature) between 59.7 °C and 60.5 °C. The RNA extraction from the BAT was performed as previously described^44^. The reverse transcription PCR reactions were performed on the QuantStudio 6 Pro Real-Time PCR system (Thermo Fisher Scientific) and quantified by the Livak method as previously described by us^44^. The data are expressed as relative mRNA abundance of the gene of interest to a housekeeping gene (*HPRT*) using the formula 2 ^(Ct(housekeeping gene) – (gene of interest))^. The exact sequences (5′–3′) of the primers (Microsynth) are as follows:

*Fasn* F: TGCCCGAGTCAGAGAACCTA

*Fasn* R:TAGAGCCCAGCCTTCCATCT

*Ndufa9* F:TCTGTCAGTGGAGTTGTGGC

*Ndufa9* R:TGTGACCCCATTCGTCCAAG

*Ucp1* F:GTGAACCCGACAACTTCCGA

*Ucp1* R: GGCCTTCACCTTGGATCTGAA

*HPRT* F: TTATCAGACTGAAGAGCTACTGAATGATC

*HPRT* R:TTACCAGTGGTCAATTGTGTCTTCAACAATC

### Statistical analysis

All data management, extraction and analysis were performed in Excel (version 2310, Microsoft Cooperation) and GraphPad Prism 10.0.3 (GraphPad Software). The values are presented as the mean ± standard deviation (s.d.) unless otherwise stated. When more than two groups were compared, either a one-way or two-way ANOVA with appropriate post hoc tests was used. One female in the accMC23 group had elephant teeth and had to be excluded from the study.

### Reporting summary

Further information on research design is available in the Nature Portfolio Reporting Summary linked to this article.

## Online content

Any methods, additional references, Nature Portfolio reporting summaries, source data, extended data, supplementary information, acknowledgements, peer review information; details of author contributions and competing interests; and statements of data and code availability are available at 10.1038/s41684-025-01618-0.

## Supplementary information

Supplementary Information Supplementary Figs. 1–8.

Reporting Summary

## Data Availability

The data that support the findings of this study are available from the corresponding author, P.S., upon reasonable request.
