# Supplementary material for: Thermoneutral environment improves mouse welfare and reduces stress in metabolic cages
Source: Lab Anim (NY). 2025 Oct 10;54(11):303–12. doi: 10.1038/s41684-025-01618-0 (PMC12575360; doi:10.1038/s41684-025-01618-0)
Supplement: Supplementary file 1 — Supplementary Figs. 1–8. [file 41684_2025_1618_MOESM1_ESM.pdf]

lab animal

Supplementary information

https://doi.org/10.1038/s41684-025-01618-0

Thermoneutral environment improves mouse welfare and reduces stress in metabolic cages

In the format provided by the authors and unedited

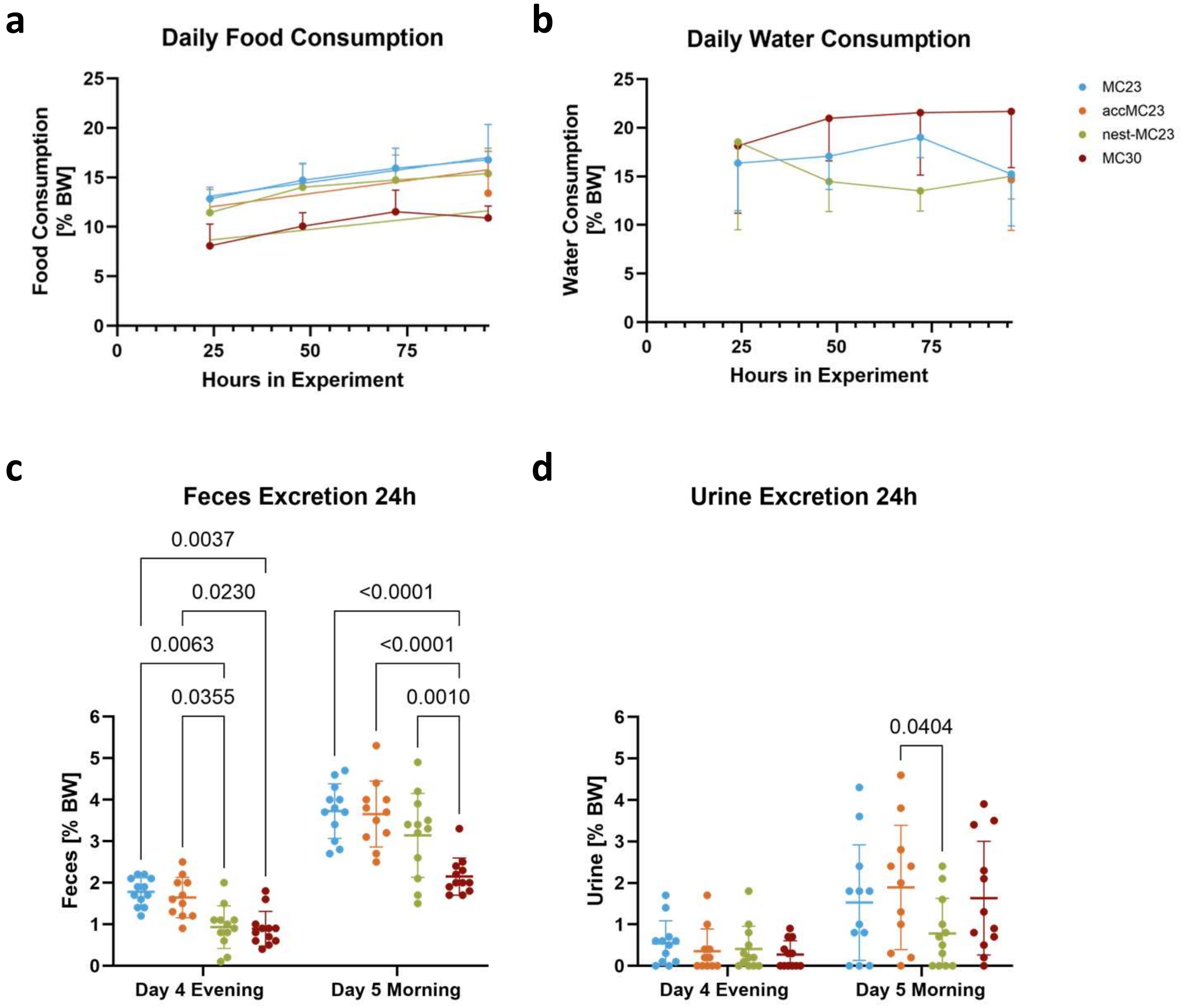

Fig. supp. 1.: Food and water consumption and urine and feces sampling during metabolic cage housing
Single-housed mice were either continuously kept in metabolic cages [MC] at 23°C for 4 days (MC23, blue, n=6♀+6♂), acclimatized for 3 h per day until day 4 (accMC23, orange, n=5♀+6♂), or provided with a nest (nest-MC23, olive, n=6♀+6♂). Another group was housed in a thermoneutral environment in the MC at 30°C (MC30, red, n=6♀+6♂). The experiment was performed in ten batches according to the availability of animals and groups. **a**, **b** Cumulative food and water intake over 24 h for all groups over the entire MC housing period. **c**, **d** Fecal and urine collection of all groups relative to their body weight at 84 h and 96 h of the MC housing period (sampling day / day 4). A two-way ANOVA with Šídák's multiple comparison test was performed (p-values indicated in graphs). For all groups and time points n = 11-12.

a

	Sodium / Creatinine (mmol/mg)/ml			Potassium / Creatinine (mmol/mg)/ml			Chloride / Creatinine (mmol/mg)/ml			Phosphorus / Creatinine (mg/mg)/ml			Urea / Creatinine (mg/mg)/ml			Magnesium / Creatinine (mg/mg)/ml			Calcium / Creatinine (mg/mg)/ml			Micro-Albumin / Creatinine (mg/mg)/dl		
	Mean	SD	RC (%)	Mean	SD	RC (%)	Mean	SD	RC (%)	Mean	SD	RC (%)	Mean	SD	RC (%)	Mean	SD	RC (%)	Mean	SD	RC (%)	Mean	SD	RC (%)
(HC23)	0.205	0.099	NA	0.376	0.072	NA	0.322	0.142	NA	3.139	1.070	-	124.900	31.400	NA	1.478	0.305	NA	0.004	0.001	NA	1.212	0.330	NA
(MC23)	0.289	0.038	40.98	0.571	0.101	51.86	0.598	0.064	85.71	6.160	1.368	96.24	226.600	26.700	81.43	1.862	0.347	25.98	0.005	0.001	25.00	1.230	0.319	1.49
(accMC23)	0.322	0.226	57.07	0.553	0.066	47.07	0.558	0.078	73.29	5.623	1.453	79.13	195.300	19.200	56.37	1.956	0.282	32.34	0.006	0.002	50.00	1.702	0.226	40.43
(nest-MC23)	0.226	0.050	10.24	0.427	0.011	13.56	0.420	0.093	30.43	4.412	0.654	40.55	169.600	17.900	35.79	1.602	0.073	8.39	0.004	0.000	0.00	0.900	0.090	-25.74
(MC30)	0.208	0.038	1.46	0.434	0.040	15.43	0.399	0.069	23.91	4.117	1.493	31.16	154.500	33.000	23.70	1.757	0.300	18.88	0.004	0.001	0.00	1.083	0.353	-10.64

b **c** **d**

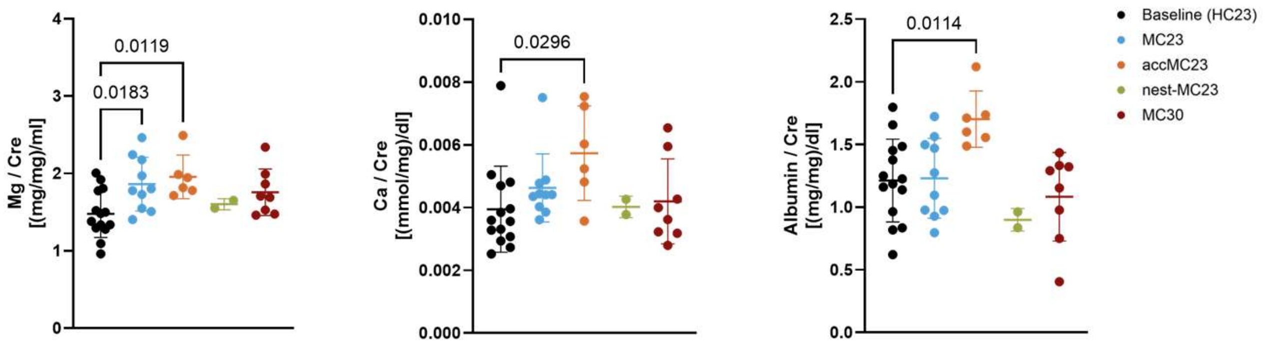

Fig. supp. 2: Urinary electrolyte and protein concentrations

Single-housed mice were either continuously kept in metabolic cages [MC] at 23°C for 4 days (MC23, blue, n=6♀+6♂), acclimatized for 3 h per day until day 4 (accMC23, orange, n=5♀+6♂), or provided with a nest (nest-MC23, olive, n=6♀+6♂). Another group was housed in a thermoneutral environment in the MC at 30°C (MC30, red, n=6♀+6♂). The experiment was performed in ten batches according to the availability of animals and groups. Male and female morning urine (7PM - 7AM) of the sampling day (day 4) was used for measurements, n = 2 - 11. Baseline samples were collected from all groups in the morning before the start of baseline measurements. The table in **a** shows the mean values (RC = relative change to baseline). Panels **b**-**d** show the concentration of magnesium (**b**), calcium (**c**), albumin (**d**) relative to creatinine excretion. One-way ANOVA with Dunnett's multiple comparison test was used for all graphs (p-values indicated in graph).

			Baseline								Metabolic Cage								Recovery							
			(MC23)		(accMC23)		(nest-MC23)		(MC30)		(MC23)		(accMC23)		(nest-MC23)		(MC30)		(MC23)		(accMC23)		(nest-MC23)		(MC30)	
			Mean	SD	Mean	SD	Mean	SD	Mean	SD	Mean	SD	Mean	SD	Mean	SD	Mean	SD	Mean	SD	Mean	SD	Mean	SD	Mean	SD
Body Core Temperature (°C)	Day	Average	36.14	0.25	36.18	0.35	36.11	0.42	36.00	0.39	35.47	0.39	36.02	0.62	35.64	0.31	35.99	0.47	36.35	0.40	36.24	0.52	36.07	0.39	36.17	0.35
		Minimum	35.15	0.31	35.25	0.36	35.24	0.46	35.08	0.40	34.56	0.47	34.81	0.52	34.77	0.34	35.31	0.62	35.04	0.37	35.16	0.53	34.77	0.83	34.97	0.42
		Maximum	37.67	0.37	37.77	0.40	37.44	0.32	37.67	0.58	37.02	0.63	37.95	0.49	37.20	0.49	37.17	0.41	38.18	0.34	38.03	0.36	38.01	0.32	37.98	0.33
	Night	Average	36.87	0.30	36.83	0.26	36.82	0.30	36.77	0.38	36.44	0.35	36.60	0.45	36.60	0.33	36.82	0.38	36.81	0.33	36.92	0.37	36.66	0.82	36.72	0.44
		Minimum	35.51	0.37	35.51	0.29	35.37	0.21	35.35	0.33	35.00	0.52	35.20	0.42	35.20	0.41	35.70	0.42	35.37	0.34	35.38	0.42	34.84	1.86	35.22	0.56
		Maximum	37.88	0.30	37.91	0.30	37.86	0.33	37.68	0.34	37.68	0.38	37.60	0.38	37.60	0.33	37.87	0.32	37.83	0.31	37.98	0.30	37.84	0.30	37.84	0.34
Heart Rate (bpm)	Day	Average	549	23	547	49	533	34	522	35	576	27	609	47	531	29	439	51	543	31	508	48	513	36	522	44
		Minimum	425	36	428	49	410	52	348	77	473	34	499	67	432	31	351	48	396	44	350	81	357	50	366	66
		Maximum	736	33	736	29	701	21	731	37	721	28	753	16	724	26	590	61	741	34	708	22	716	34	720	38
	Night	Average	587	30	579	37	577	21	554	37	627	28	641	22	582	23	496	32	561	27	536	40	545	40	550	41
		Minimum	418	46	409	44	403	52	348	71	501	45	496	42	405	28	363	42	392	41	358	55	361	45	379	45
		Maximum	737	30	731	28	725	23	731	26	753	28	749	22	744	29	651	30	724	18	705	23	713	42	726	29
Respiratory Rate (bpm)	Day	Average	161	8	160	9	164	14	154	7	166	10	174	12	159	14	126	11	163	12	161	17	155	13	154	11
		Minimum	126	10	127	7	128	19	100	33	137	11	146	16	133	10	105	9	124	20	111	35	111	17	115	14
		Maximum	207	14	205	11	204	13	199	12	211	11	209	7	210	16	161	16	206	14	212	21	199	18	201	9
	Night	Average	175	7	176	8	181	14	168	8	190	10	188	11	184	11	144	8	171	11	169	16	169	15	166	9
		Minimum	136	9	140	9	142	13	118	23	153	15	147	12	142	12	115	8	132	16	124	27	128	17	128	13
		Maximum	217	9	219	9	223	20	214	10	233	10	226	10	230	14	189	11	216	15	222	18	211	17	210	13
Mean Arterial Bloodpressure (mmHg)	Day	Average	90.54	4.87	92.67	4.04	92.38	3.89	93.79	8.86	92.65	4.92	96.31	4.07	95.32	5.33	87.23	5.10	91.35	5.85	90.30	4.41	92.82	4.74	92.92	7.46
		Minimum	74.74	5.90	76.46	2.34	76.96	4.14	76.01	6.13	80.38	5.39	82.49	5.48	81.59	4.48	74.34	4.33	73.73	7.63	73.99	6.80	73.72	7.33	71.11	6.55
		Maximum	111.17	8.42	118.35	15.91	112.34	10.02	121.72	17.55	110.62	8.53	116.99	5.55	116.63	11.02	109.08	12.24	114.16	9.52	114.39	7.57	118.26	6.29	120.62	19.46
	Night	Average	96.91	4.39	99.67	6.32	98.90	4.20	102.68	9.60	99.93	5.00	106.24	6.64	103.76	4.34	94.98	8.37	95.69	7.25	97.35	6.20	98.91	5.15	99.22	7.62
		Minimum	77.51	4.04	77.15	5.02	77.89	5.81	80.60	7.36	84.47	5.81	85.24	10.02	83.31	3.79	77.02	4.08	76.84	6.79	74.73	5.85	75.63	7.88	75.44	5.88
		Maximum	112.63	8.35	118.85	16.44	116.99	12.36	125.40	20.08	114.29	8.19	120.44	9.13	121.90	7.49	114.09	16.80	113.10	12.50	116.81	8.62	120.55	8.06	121.71	18.66

Fig. supp. 3.: Absolute values for vital parameters

Single-housed mice were either kept in metabolic cages [MC] at 23°C for 4 days continuously (MC23, blue, n=6♀+6♂), acclimated for 3h per day until day 4 (accMC23, orange, n=5♀+6♂) or provided with a nest (nest-MC23, olive, n=6♀+6♂). Another group was housed in a thermoneutral environment in the MC at 30°C (MC30, red, n=6♀+6♂). The experiment was performed in ten batches, depending on the availability of animals and groups. The table shows the mean ± SD, minimum ± SD and maximum ± SD of the absolute values for all groups during the baseline (0-48h), MC housing (only sampling day / day 4) and recovery (144-216h) periods. Furthermore, the values are separated for the light-on (7am-7pm) and light-off (7pm-7pm) periods. The vital parameters were measured with an abdominally implanted HD-S11 transmitter and the recording rate was set to a 10-minute interval. All implanted mice were used to calculate mean, maximum and minimum values, except for blood pressure data due to missing blood pressure data (n = 10-12).

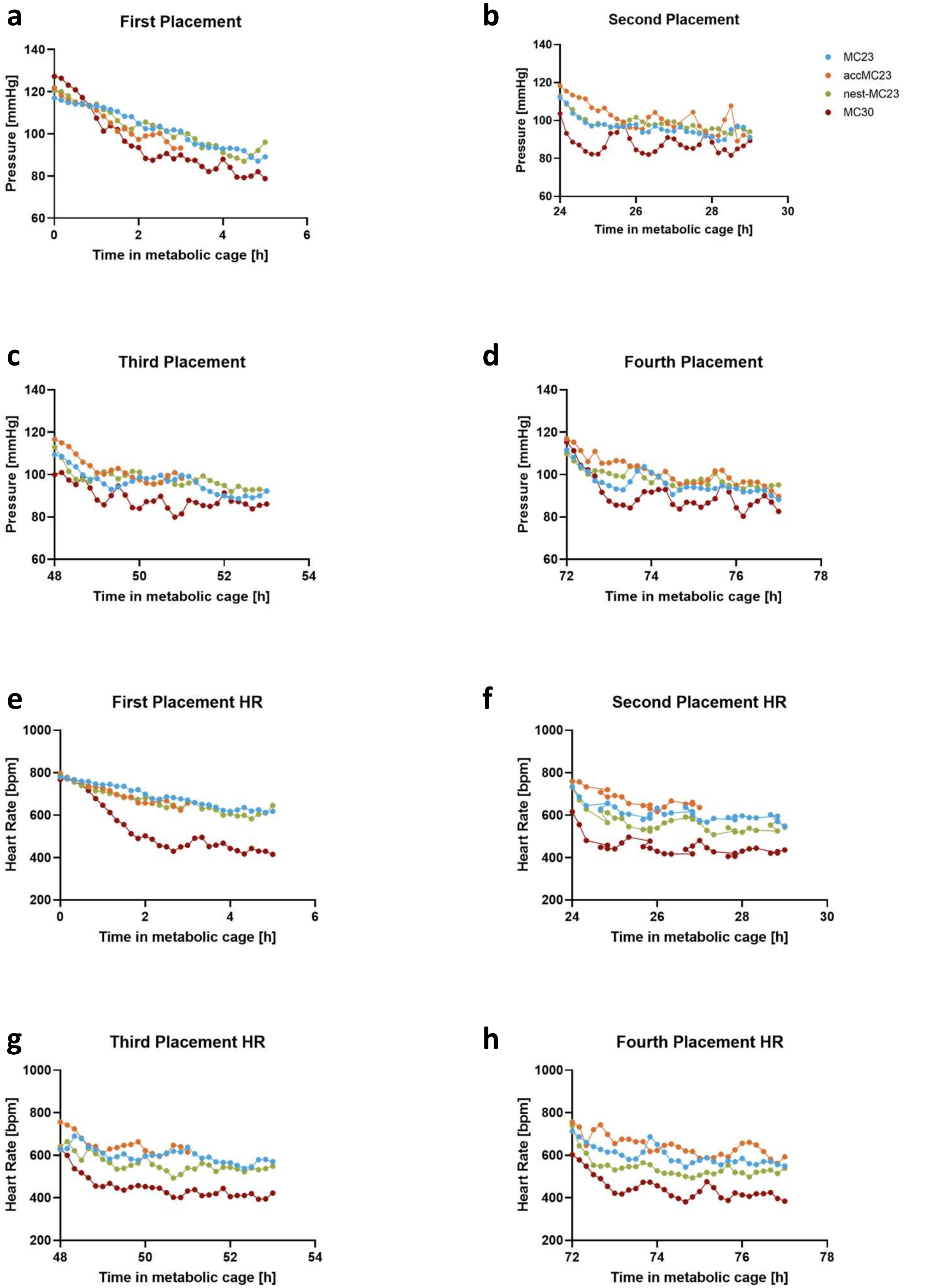

Fig. supp. 4.: Blood pressure and heart rate after placement in the metabolic cage
Single-housed mice were either kept in metabolic cages [MC] at 23°C for 4 days continuously (MC23, blue, n=6♀+6♂), acclimated for 3h per day until day 4 (accMC23, orange, n=5♀+6♂), or provided with a nest (nest-MC23, olive, n=6♀+6♂). Another group was housed in a thermoneutral environment in the MC at 30°C (MC30, red, n=6♀+6♂). The experiment was performed in ten batches, depending on the availability of animals and groups. **a**-**d** Average of mean arterial blood pressure each morning after animals were returned to the MC after handling, or after handling with cage change for the accMC23 group. In **e**-**h** heart rate is shown for the same period. The parameters were measured with an abdominally implanted HD-S11 transmitter.

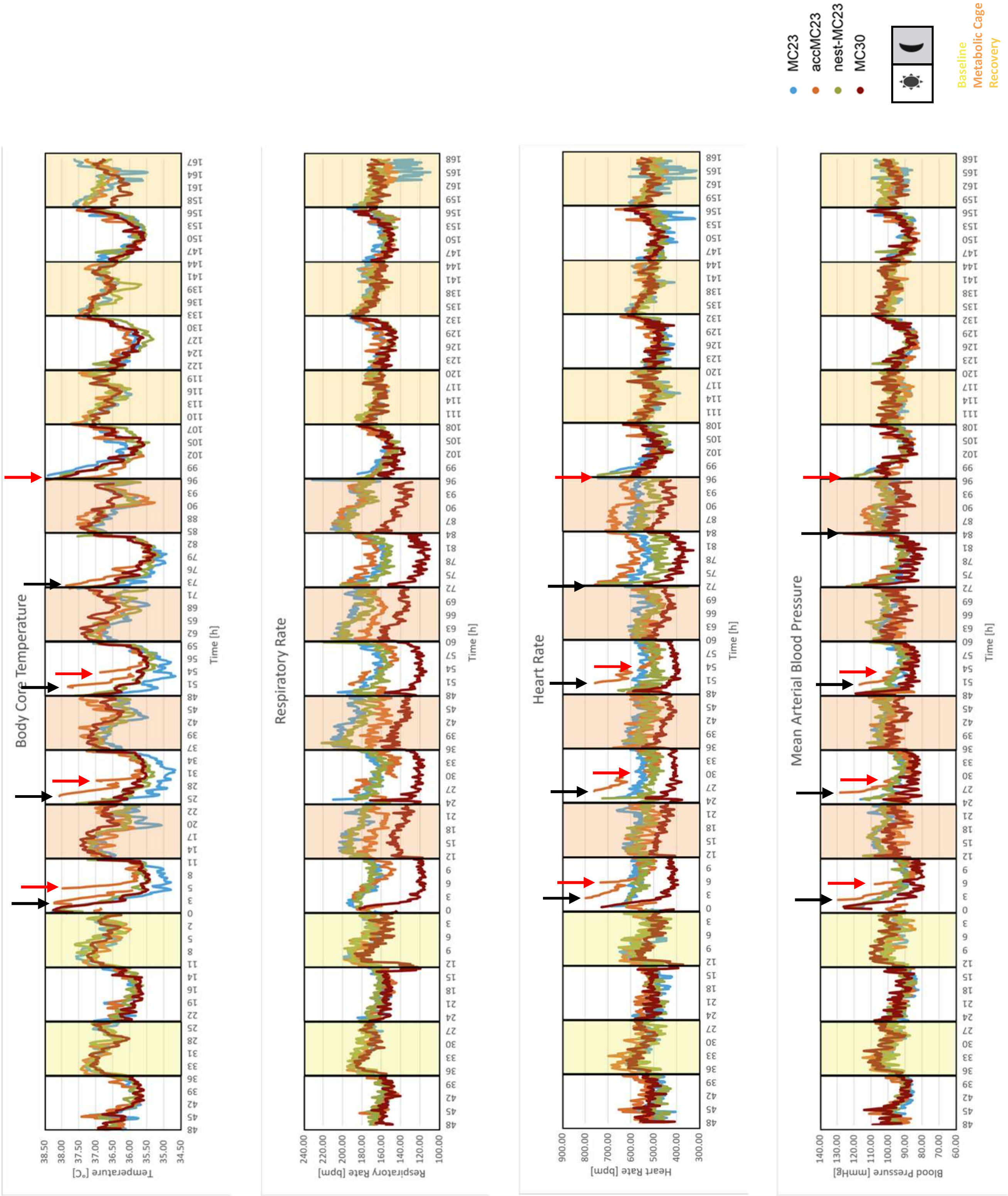

Fig. supp. 5.: Average vital parameters throughout the experiment

Single-housed mice were either continuously kept in metabolic cages [MC] at 23°C for 4 days (MC23, blue, n=6♀+6♂), acclimatized for 3 h per day until day 4 (accMC23, orange, n=5♀+6♂), or provided with a nest (nest-MC23, olive, n=6♀+6♂). Another group was housed in a thermoneutral environment in the MC at 30°C (MC30, red, n=6♀+6♂). **a**-**d** Average of core body temperature (**a**), respiratory rate (**b**), heart rate (**c**) and mean arterial blood pressure (**d**). The first 48h show the baseline parameters in the home cage, followed by the 96h of metabolic cage housing (according to groups: MC23, blue; accMC23, orange; nest-MC23, olive; MC30, red) and the 72h recovery period in the home cage. The black arrows indicate when the mice were placed in the MC (coming from the home cage), whereas the red arrows indicate when the mice were placed in the home cage (after handling, i.e. health check). Vital signs were measured using an abdominally implanted HD-S11 transmitter. For all groups n=10-12.

		(accMC23)						(MC23)			
		Day1 - Day3 Home Cage		Day1 - Day3 Metabolic Cage		Sampling Day		Day1 - Day3 Metabolic Cage		Sampling Day	
		Mean	SD	Mean	SD	Mean	SD	Mean	SD	Mean	SD
Body Core Temperature (°C)	Average	35.99	0.45	36.51	0.38	36.34	0.46	35.45	0.49	35.47	0.39
	Minimum	35.02	0.48	34.92	0.50	34.41	0.43	34.03	0.64	34.56	0.47
	Maximum	37.77	0.36	38.36	0.15	38.08	0.28	38.20	0.30	37.02	0.63
Heart Rate (bpm)	Average	526	42	553	33	625	31	608	29	576	27
	Minimum	403	40	373	32	475	44	479	45	473	34
	Maximum	720	36	785	19	760	16	775	21	721	28
Respiratory Rate (bpm)	Average	155	9	166	8	181	10	171	8	166	10
	Minimum	126	8	124	10	142	11	134	11	137	11
	Maximum	195	11	214	9	226	10	227	13	211	11
Mean Arterial Bloodpressure (mmHg)	Average	89.53	3.39	95.12	3.13	101.48	4.56	94.85	5.15	92.65	4.92
	Minimum	74.49	6.38	73.152	6.31	81.02	7.49	79.27	5.92	80.38	5.39
	Maximum	111.94	7.69	123.46	9.70	120.85	8.84	117.34	7.38	110.62	8.53

Fig. supp. 6.: Absolute values of vital parameters compared between accMC23 and MC23 groups
Single-housed mice were either continuously kept in metabolic cages [MC] at 23°C for 4 days (MC23, n=6♀+6♂) acclimatized for 3 h per day until day 4 (accMC23, n=5♀+6♂). The period is divided into days 1-3, which were the acclimatization days of the accMC23 group (3h metabolic cage and 21h home cage), and day 4, when both groups were housed in MCs for 24h. Vital parameters were measured with an abdominally implanted HD-S11 transmitter and the recording rate was adjusted to a 10 min interval. All implanted mice were used to calculate mean, maximum and minimum values, except for blood pressure data due to missing data (n = 10-12).

a

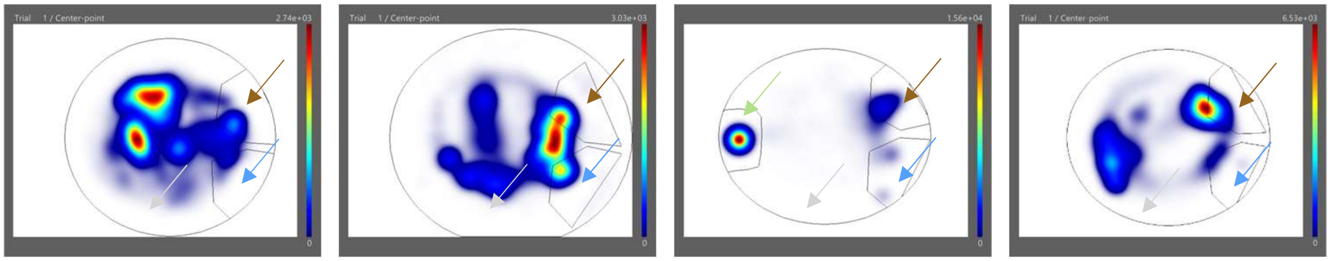

b

	% of Time in Food Zone		% of Time in Water Zone		% of Time in Nest Zone		% of Time on the Grid	
	Day	Night	Day	Night	Day	Night	Day	Night
**(MC23)**	10.29	18.93	7.42	3.47	NA	NA	79.62	73.69
**(accMC23)**	6.70	15.52	1.74	4.76	NA	NA	87.15	74.39
**(nest-MC23)**	4.02	14.26	0.38	2.09	89.70	67.46	1.74	7.92
**(MC30)**	6.91	11.73	2.82	2.12	NA	NA	87.85	83.23

Fig. supp. 7.: Exact number of relative time spent in specific zones during metabolic cage housing
Single-housed mice were either continuously kept in metabolic cages [MC] at 23°C for 4 days (MC23, blue, n=6♀+6♂), acclimated for 3 h per day until day 4 (accMC23, orange, n=5♀+6♂), or provided with a nest (nest-MC23, olive, n=6♀+6♂). Another group was housed in a thermoneutral environment in the MC at 30°C (MC30, red, n=6♀+6♂). The experiment was performed in ten batches, depending on the availability of animals and groups. **a** Heat map showing the zone (food zone: brown arrow, water zone: blue arrow, grid zone: gray arrow, nest zone: green arrow) of the tracking arena in which the mouse spent most of its time. The heat maps always show one representative individual for each housing condition (from left to right: MC23, accMC23, nest-MC23, MC30). **b** Mean relative time spent in each zone for all groups on the sampling day in the MCs.

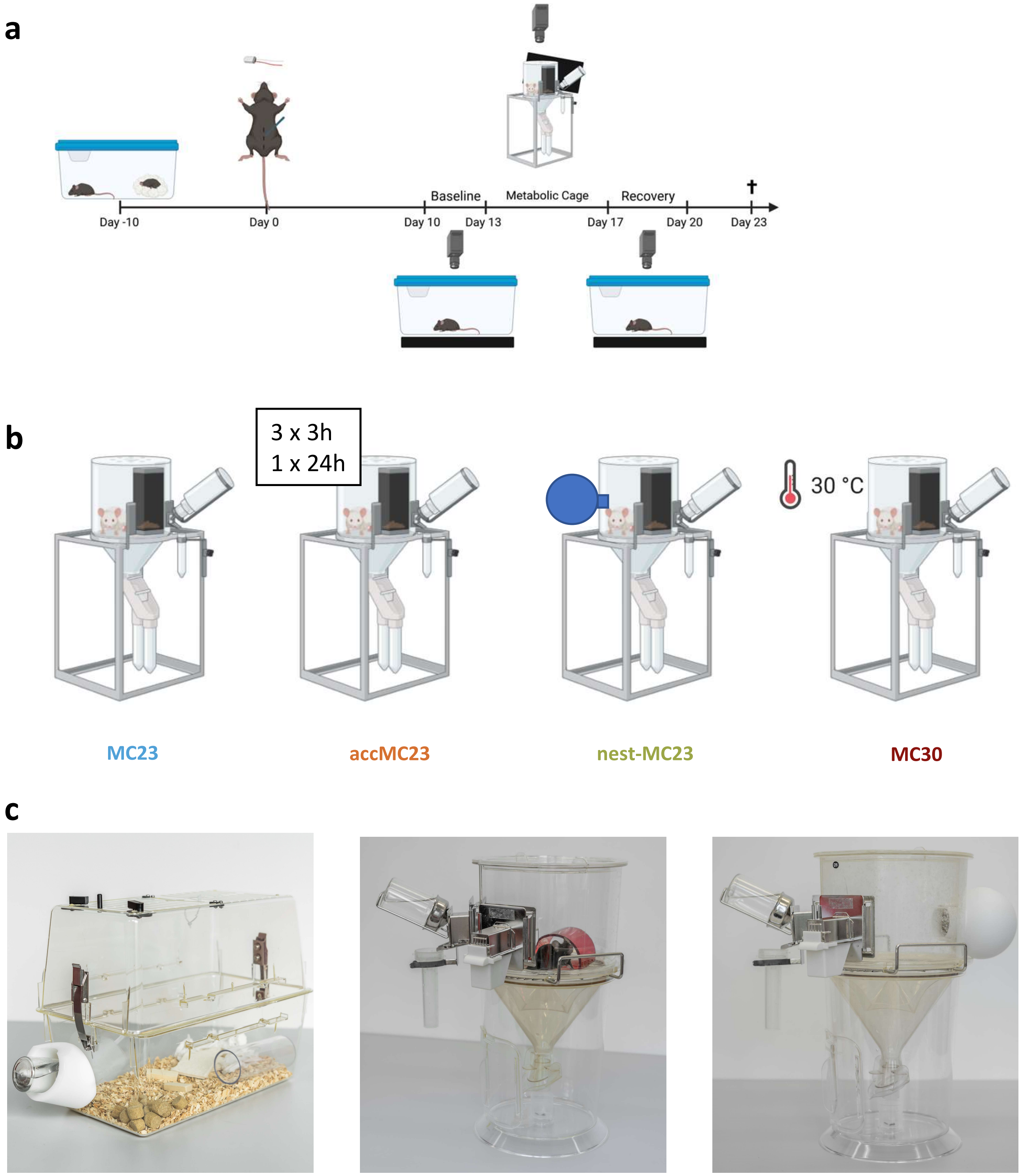

Fig. supp. 8.: Experimental timeline, groups and housing conditions

a Experimental setup in a timeline, on day -10 or earlier, mice arrived at the facility and underwent surgery on day 0. After 10 days of recovery, baseline was measured for 48h, followed by metabolic cage housing for 96h (divided into days 1-3 and the sampling day (day 4)), mice were then moved to their home cage for 72h of recovery. Earliest euthanasia was on day 23. **b** The four different experimental groups: at 23°C for 96h MC23, at 23°C for 3x3h and 24h accMC23, provided with a nest and nesting material nest-MC23 and in a thermoneutral environment MC30. **c** home cage (left), standard MC (middle) and a MC equipped with the 3D printed shelter (right). Mice were single-housed throughout the experiment (except surgical recovery phase). **a**-**b** “created with Biorender”
