# Supplementary material for: Thermoneutral environment improves mouse welfare and reduces stress in metabolic cages
Source: Lab Anim (NY). 2025 Oct 10;54(11):303–12. doi: 10.1038/s41684-025-01618-0 (PMC12575360; doi:10.1038/s41684-025-01618-0)
Supplement: Supplementary file 2 — Reporting Summary [file 41684_2025_1618_MOESM2_ESM.pdf]

nature portfolio

Corresponding author(s): Dr. Petra Seebeck

Last updated by author(s): Jan 30, 2025

Reporting Summary

Nature Portfolio wishes to improve the reproducibility of the work that we publish. This form provides structure for consistency and transparency in reporting. For further information on Nature Portfolio policies, see our Editorial Policies and the Editorial Policy Checklist.

Statistics

For all statistical analyses, confirm that the following items are present in the figure legend, table legend, main text, or Methods section.

n/a	Confirmed	
☐	☒	The exact sample size (*n*) for each experimental group/condition, given as a discrete number and unit of measurement
☐	☒	A statement on whether measurements were taken from distinct samples or whether the same sample was measured repeatedly
☐	☒	The statistical test(s) used AND whether they are one- or two-sided<br>*Only common tests should be described solely by name; describe more complex techniques in the Methods section.*
☐	☒	A description of all covariates tested
☐	☒	A description of any assumptions or corrections, such as tests of normality and adjustment for multiple comparisons
☐	☒	A full description of the statistical parameters including central tendency (e.g. means) or other basic estimates (e.g. regression coefficient) AND variation (e.g. standard deviation) or associated estimates of uncertainty (e.g. confidence intervals)
☐	☒	For null hypothesis testing, the test statistic (e.g. *F*, *t*, *r*) with confidence intervals, effect sizes, degrees of freedom and *P* value noted<br>*Give P values as exact values whenever suitable.*
☒	☐	For Bayesian analysis, information on the choice of priors and Markov chain Monte Carlo settings
☒	☐	For hierarchical and complex designs, identification of the appropriate level for tests and full reporting of outcomes
☒	☐	Estimates of effect sizes (e.g. Cohen's *d*, Pearson's *r*), indicating how they were calculated

Our web collection on statistics for biologists contains articles on many of the points above.

Software and code

Policy information about availability of computer code

Data collection	Ponemah software 6.51 (Data Science International, USA); media recorder software 6.2 (Noldus, The Netherlands)
Data analysis	Ponemah software 6.51 (Data Science International, USA); CapCut 2.7.0 (ByteDance, China); EthoVision XT 17.5 (Noldus, The Netherlands); GraphPad Prism 10.0.3(GraphPad Software LLC, USA)

For manuscripts utilizing custom algorithms or software that are central to the research but not yet described in published literature, software must be made available to editors and reviewers. We strongly encourage code deposition in a community repository (e.g. GitHub). See the Nature Portfolio guidelines for submitting code & software for further information.

Data

Policy information about availability of data

All manuscripts must include a data availability statement. This statement should provide the following information, where applicable:

- Accession codes, unique identifiers, or web links for publicly available datasets
- A description of any restrictions on data availability
- For clinical datasets or third party data, please ensure that the statement adheres to our policy

The data that support the findings of this study are available on request from the corresponding author [PS, CAW].

March 2021

Field-specific reporting

Please select the one below that is the best fit for your research. If you are not sure, read the appropriate sections before making your selection.

☒ Life sciences ☐ Behavioural & social sciences ☐ Ecological, evolutionary & environmental sciences

For a reference copy of the document with all sections, see nature.com/documents/nr-reporting-summary-flat.pdf

Life sciences study design

All studies must disclose on these points even when the disclosure is negative.

Sample size	Group size was n=12 (6 females and 6 males)<br>Group size may changes depending on analysis (e.g. blood pressure related data, due malfunctioning blood pressure catheter or missing urinary samples). If the number of experimental unites changed it is indicated in the plot (scatter plot) and/or the figure legend
Data exclusions	One female mouse from the accMC23 group was excluded as stated in the manuscript. Reason: elephant teethes who changed the food intake pattern and presumably the stress levels.
Replication	We did not exactly replicate a specific experiment or setup, but main messages of previous studies could be confirmed. Methods are described as detailed as possible to allow for replication of our findings.
Randomization	Randomization on the individual mouse level was not possible since batches of mice had to be used at once (logistical limitations of the hardware availability and mice availability because of surgery success).
Blinding	The investigators have not been blinded during the experiment itself since the blinding was impossible due to the experimental setup (house, temperature,...).

Reporting for specific materials, systems and methods

We require information from authors about some types of materials, experimental systems and methods used in many studies. Here, indicate whether each material, system or method listed is relevant to your study. If you are not sure if a list item applies to your research, read the appropriate section before selecting a response.

Materials & experimental systems

n/a	Involved in the study	
☒	☐	Antibodies
☒	☐	Eukaryotic cell lines
☒	☐	Palaeontology and archaeology
☐	☒	Animals and other organisms
☒	☐	Human research participants
☒	☐	Clinical data
☒	☐	Dual use research of concern

Methods

n/a	Involved in the study	
☒	☐	ChIP-seq
☒	☐	Flow cytometry
☒	☐	MRI-based neuroimaging

Animals and other organisms

Policy information about studies involving animals; ARRIVE guidelines recommended for reporting animal research

Laboratory animals	14 - 20 week old wild type C57BL6/N mice of both sexes (males and females) obtained from Charles River (Germany) were used for the experiment. All details regarding numbers and animal housing conditions are described in the methodological part of the submitted paper.
Wild animals	There were no wild animals used for this study.
Field-collected samples	There were no field-collected samples used for this study.
Ethics oversight	All animal experiments were designed according to the PREPARE/ARRIVE guidelines and approved by the Swiss cantonal veterinary authorities (licence numbers ZH212/2021 and ZH129/2021).

Note that full information on the approval of the study protocol must also be provided in the manuscript.
